# In-hospital experience with insulin degludec (IDeg)

**DOI:** 10.1186/1758-5996-7-S1-A90

**Published:** 2015-11-11

**Authors:** Maria Gabriela Pedigoni Bulisani, Maria Fernanda Ozorio de Almeida, Camila Miranda Abdon, Ana Cláudia Souza Moreno, Marília Izar Helfenstein Fonseca, Paulo Roberto Rizzo Genestreti

**Affiliations:** 1 Santa Casa, São Paulo, Brazil

## Background

Glycemic control is critical for in-patients and dysglycemia is associated with worse prognosis and higher mortality. Insulin therapy is considered the best treatment on this scenario and basal insulin analogues, such as insulin glargine (IGlar) and IDeg, could be useful options, however there are no studies comparing IGlar and IDeg evaluating glucose variability (GV) in hospitalized patients, neither the transition between them.

## Objective

To present a case series describing the efficacy and safety of IDeg in-patientes with diabetes using GV and rate of hypoglycemia when compared with IGlar.

## Materials and methods

Retrospective analysis of blood glucose obtained with point-of-care testing of 10 diabetic patients admitted at Bandeirantes Hospital, Sao Paulo, between October 2014 and April 2015, previously treated with IGlar for a minimum of 7 days and switched to IDeg for at least 7 days more during hospitalization. Parameters studied included GV, standard deviation (SD), coefficient of variation (CV) and mean glucose levels, obtained from software PXP Abbott. Hypoglycemia was defined as blood glucose (BG) <70mg/dL and it was severe if BG <40mg/dL. Basal insulin dose was compared on the last day of both, after achievement of steady state.

## Results

**Figure 1:**
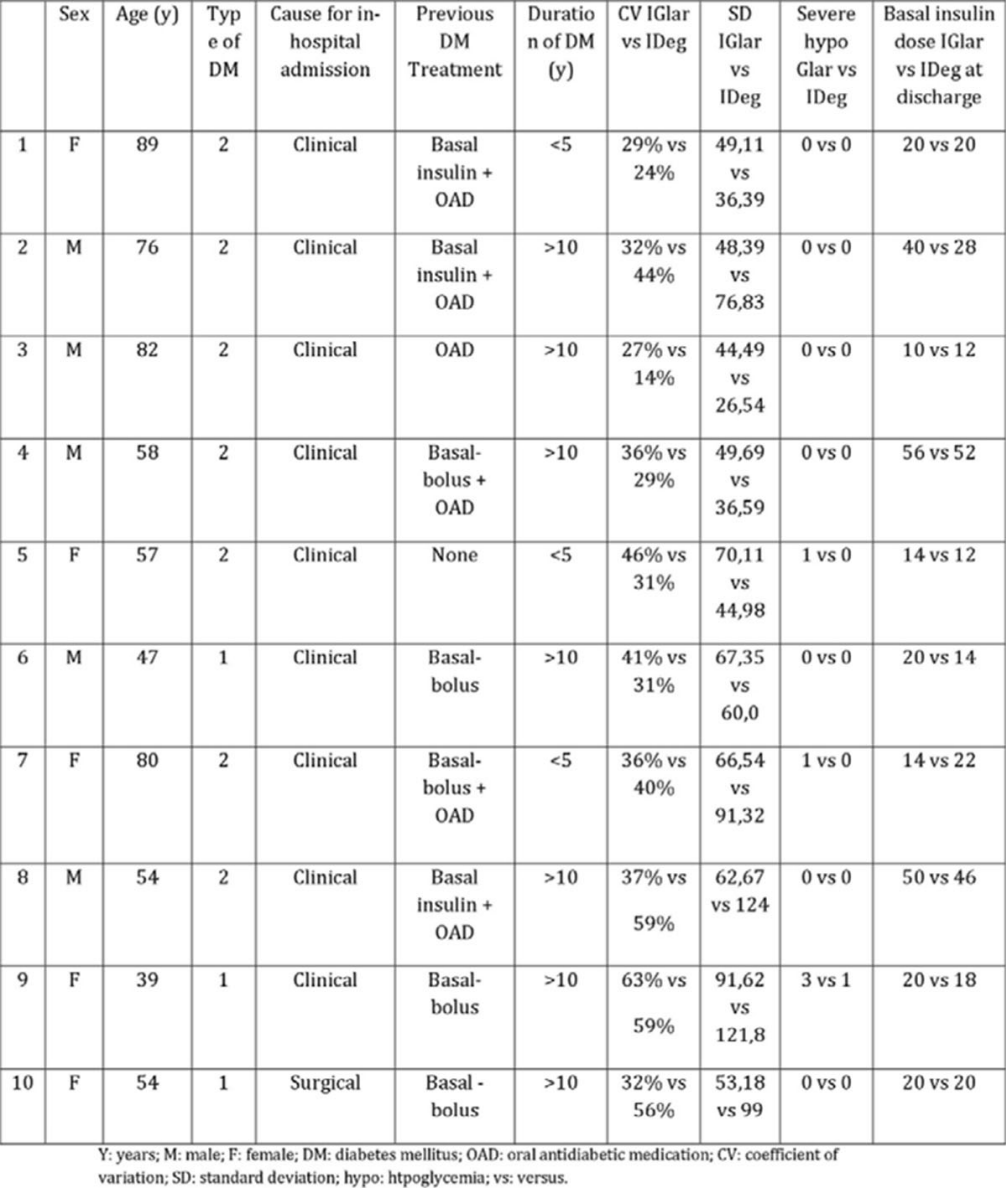
Summary of results

3/10 patients had type 1 diabetes mellitus (T1D) for over 10 yrs., previous treatment with insulin therapy in a basal-bolus regimen. Average age was 46 yrs. and mean HbA1c was 9% and no benefit could be noticed. 7/10 patients had type 2 diabetes mellitus (T2D), with duration of >10 yrs., the majority had previous insulin treatment. Mean age was 70 yrs. and mean HbA1c was 9,7%. All T2D patients maintained CV, and 57% had a reduction in SD, improving GV. Basal insulin dose with IDeg was lower at discharge as compared to IGlar in T2D. Severe hypoglycemia events were diminished after switching (see Figure 1).

## Conclusions

In this report, GV was lower in T2D patients treated with IDeg as compared to IGlar, although the same could not be seen in T1D, perhaps due to the small number of patients included. More studies in this population are needed to confirm this hypothesis and continuous glucose monitoring should be preferred. IDeg proved to be a safe and effective alternative at hospital and might improve GV. This would be better due to its predictability of effect and low rate of hypoglycemia, important among hospitalized patients.

